# Preliminary validation of the pica, ARFID and rumination disorder interview ARFID questionnaire (PARDI-AR-Q)

**DOI:** 10.1186/s40337-022-00706-7

**Published:** 2022-11-22

**Authors:** Rachel Bryant-Waugh, Casey M. Stern, Melissa J. Dreier, Nadia Micali, Lucy J. Cooke, Megan C. Kuhnle, Helen Burton Murray, Shirley B. Wang, Lauren Breithaupt, Kendra R. Becker, Madhusmita Misra, Elizabeth A. Lawson, Kamryn T. Eddy, Jennifer J. Thomas

**Affiliations:** 1 South London and Maudsley NHS Foundation Trust, London, UK; 2 Institute of Psychiatry, Psychology and Neuroscience, Kings College London, London, UK; 3 Eating Disorders Clinical and Research Program, Massachusetts General Hospital, 2 Longfellow Place, Suite 200, Boston, MA 02114 USA; 4 Department of Psychology, Rutgers University, Piscataway, USA; 5 Department of Psychiatry, University of Geneva, Geneva, Switzerland; 6 Department of Pediatrics, Gynecology and Obstetrics, University of Geneva, Geneva, Switzerland; 7 Eating Disorders Research Unit, Mental Health Center Ballerup, Mental Health Services of the Capital Region of Denmark, Ballerup, Denmark; 8 Great Ormond Street Hospital for Children NHS Trust, London, UK; 9 Department of Epidemiology, Boston University, Boston, USA; 10 Center for Neurointestinal Health, Massachusetts General Hospital, Boston, USA; 11 Harvard Medical School, Boston, USA; 12 Department of Psychology, Harvard University, Cambridge, USA; 13 Department of Psychiatry, Athinoula A. Martinos Center for Biomedical Imaging, Boston, USA; 14 Neuroendocrine Unit, Massachusetts General Hospital, Boston, USA

## Abstract

**Background:**

The Pica, ARFID, and Rumination Disorder Interview (PARDI) is a structured interview that can be used to determine diagnosis, presenting characteristics, and severity across three disorders, including avoidant/restrictive food intake disorder (ARFID). The purpose of this study was to evaluate the psychometric properties of a questionnaire focused specifically on ARFID (PARDI-AR-Q), which has the potential to provide related information with less participant burden.

**Methods:**

Adolescents and adults (*n* = 71, ages 14–40 years) with ARFID (*n* = 42) and healthy control participants (HC, *n* = 29) completed the PARDI-AR-Q and other measures. A subset of the ARFID group (*n* = 27) also completed the PARDI interview.

**Results:**

An exploratory factor analysis of proposed subscale items identified three factors corresponding to the ARFID phenotypes of avoidance based on the sensory characteristics of food, lack of interest in eating or food, and concern about aversive consequences of eating. Further analyses supported the internal consistency and convergent validity of the PARDI-AR-Q subscales, and subscale ratings on the questionnaire showed large and significant correlations (all *p*-values < 0.001; *r*’s ranging from 0.48 to 0.77) with the corresponding subscales on the interview. The ARFID group scored significantly higher than HC on all subscales. Furthermore, 90% of the ARFID group scored positive on the PARDI-AR-Q diagnostic algorithm while 93% of the HC scored negative.

**Conclusions:**

Though replication in larger and more diverse samples is needed, findings provide early support for the validity of the PARDI-AR-Q as a self-report measure for possible ARFID in clinical or research settings.

**Supplementary Information:**

The online version contains supplementary material available at 10.1186/s40337-022-00706-7.

## Plain English summary

Few measures are available for detecting avoidant/restrictive food intake disorder (ARFID) in clinics or research settings. This makes it difficult for individuals with the disorder to be identified and offered appropriate care. We developed the Pica, ARFID, and Rumination Disorder Interview ARFID Questionnaire (PARDI-AR-Q), a brief self-report measure of possible ARFID, and evaluated how well it worked in the current study. In our preliminary study of 71 adolescents and adults, most individuals with ARFID (90%) scored positive on this measure, whereas most healthy control participants (93%) scored negative. Our findings provide early support for the PARDI-AR-Q as a promising new measure for detecting possible ARFID when a full clinical interview is not possible.

**Supplementary Information:**

The online version contains supplementary material available at 10.1186/s40337-022-00706-7.

## Introduction

Although avoidant/restrictive food intake disorder (ARFID) was added to the psychiatric nomenclature nearly 10 years ago, there are still few available measures designed to identify potential cases and assess their clinical features [1]. Widely used self-report questionnaires for eating disorders—such as the Eating Disorder Examination-Questionnaire [2], Eating Attitudes Test [3], Eating Disorders Inventory [4] and Eating Pathology Symptoms Inventory [5]—focus primarily on purposeful dietary restriction in the context of shape/weight concerns, which is an exclusion criterion for ARFID. Available structured clinical interviews that include ARFID criteria either have not been validated for ARFID specifically (e.g., Eating Disorder Assessment for *DSM-5* [6]; Structured Clinical Interview for *DSM-5* [7]) or are lengthy and may not be practical for routine use in clinical settings (e.g., Pica, ARFID, and Rumination Disorder Interview [8]; Eating Disorder Examination ARFID module [9]). Available self-report measures of ARFID symptoms include the Nine-Item ARFID Screen (NIAS) [10], which provides data on the three ARFID phenotypes that may motivate food avoidance and restriction in ARFID (including subscales labeled “picky eating,” “fear,” and “low appetite”). However, screening positive or negative for ARIFD on the NIAS has not been validated against diagnosis via clinical interview, and cut-points for a possible ARFID diagnosis have only recently been proposed [11]. Thus, a brief questionnaire that elucidates possible ARFID in both clinical and research settings, and summarizes clinical features that may be relevant to treatment planning (e.g., selecting modules to apply according to ARFID phenotype [12, 13]) is urgently needed.

To that end, the purpose of the current study was to provide a preliminary evaluation of the construct validity and reliability of the Pica, ARFID and Rumination Disorder Interview ARFID Questionnaire (PARDI-AR-Q). Just as the EDE-Q [2] is a brief questionnaire assessing similar constructs to those evaluated in greater depth on the EDE interview [14], the PARDI-AR-Q is a brief questionnaire assessing similar constructs to those evaluated in the PARDI. The PARDI-AR-Q focuses on the psychopathology of ARFID specifically and does not include items keyed to pica or rumination disorder. By inviting adolescents and adults with ARFID, as well as healthy control participants (HC), to complete the PARDI-AR-Q, we tested the following hypotheses. First, using an exploratory factor analysis of this new measure in the ARFID group only, we anticipated that we would identify three factors consistent with the three ARFID phenotypes described in *DSM-5* (avoidance based on sensory characteristics of food, lack of interest, concern about aversive consequences [15]), each of which would exhibit a high degree of internal consistency. Second, across the whole sample, we predicted that three factors, as well as a severity of impact scale measuring the clinical impairment associated with ARFID symptoms, would correlate strongly and significantly with related constructs (supporting convergent validity) but not with unrelated constructs (supporting divergent validity). We further explored, in a subset of ARFID participants who also completed the PARDI interview, whether the PARDI-AR-Q would show a high degree of concurrent validity with subscales measuring similar constructs on the interview. Third, we predicted that, compared to HC, individuals with ARFID would score significantly higher on all subscales (avoidance based on sensory characteristics of food, lack of interest, concern about aversive consequences, and severity of impact), and that the majority of the ARFID group would score positive on the PARDI-AR-Q diagnostic algorithm while the majority of HC would score negative.

## Method

### Participants

Table 1 provides demographic characteristics of the sample. We recruited participants with ARFID (*n* = 42, ages 14–40 years) from two NIMH-funded studies (R01MH108595, investigating the neurobiology of ARFID; and R01MH103402, examining low-weight eating disorders), as well as consecutive referrals to the Eating Disorders Clinical and Research Program at the Massachusetts General Hospital. Neurobiology study participants were diagnosed with ARFID via the PARDI [8], and clinic patients were diagnosed with ARFID via routine clinical interview by their treating psychologist or psychiatrist according to *DSM-5* criteria [16]. Because the purpose of the current study was to evaluate the performance of the PARDI-AR-Q in identifying full-threshold cases of ARFID, only individuals diagnosed with full-threshold ARFID via clinical interview were included; we excluded subthreshold cases. Furthermore, given that the PARDI-AR-Q is meant for ages 14 and up, we excluded participants below the age of 14 from both recruitment sources. We excluded individuals over the age of 40 as there were none in the neurobiology study and very few in the clinic sample.

**Table 1:** Demographic characteristics of the avoidant/restrictive food intake disorder (ARFID) group and healthy control participants (HC)

	ARFID *n* = 42	HC *n* = 29*	Test statistic (*X*^2^*,t*)	*p*	Effect size (*V, d*)
Sex (n [% female])	23 [55%]	8 [28%]	4.10	0.04	0.27
Age (years; M [SD])	19.3 [3.7]	29.9 [7.6]	− 6.28	< 0.001	0.70
Race (*n* [%])			7.50	0.06	0.33
American Indian/Alaska native	0 [0%]	0 [0%]			
Asian	0 [0%]	2 [7%]			
Black or African American	0 [0%]	2 [7%]			
More than one race	2 [5%]	2 [7%]			
Native Hawaiian or Pacific Islander	0 [0%]	0 [0%]			
White	40 [95%]	22 [81%]			
Ethnicity (*n* [%])			0.01	0.92	0.08
Non-hispanic	39 [93%]	28 [100%]			
Hispanic	3 [7%]	1 [4%]			
BMI percentile/absolute BMI (M [SD])					
BMI percentile for ages 14–17 years	41.9 [30.6]	51.0 [24.8]	− 0.67	0.52	0.31
Absolute BMI (kg/m^2^) for ages ≥ 18 years	23.3 [7.3]	28.1 [7.5]	− 2.79	0.007	0.70

*BMI* body mass index

*n = 1 participant declined to provide race and ethnicity data. Race and ethnicity statistics for HC were calculated without this participant (i.e., *n* = 28)

We recruited HC (*n* = 29, ages 14–40 years, to match the age range of the ARFID group) who had either served as healthy controls from the neurobiology study (*n* = 1), learned about the study from Rally (a Mass General Brigham-wide recruitment platform; *n* = 4), or participated via Amazon Mechanical Turk (MTurk, an online recruitment platform for survey research; *n* = 24). Healthy controls from the neurobiology study did not meet criteria for any current psychiatric disorder on the Kiddie Schedule for Affective Disorders and Schizophrenia (KSADS) [17]. Adults and children from MTurk and Rally were classified as HC through a self-report battery; they had to score below clinical cut points including < 2.3 on the Eating Disorder Examination-Questionnaire [2], < 44 on the State–Trait Anxiety Inventory trait scale [18], < 16 on the Center for Epidemiological Studies Depression Scale [19], and < 10, 9, and 10, respectively, on the Picky Eating, Appetite, and Fear subscales of the Nine-Item ARFID Scale [10, 11]. To ensure data quality for individuals recruited through MTurk, we set our survey such that individuals could not participate twice. Additionally, and in line with recommendations for analyzing MTurk data [20], we ensured there were no duplicate entries by collecting MTurk worker IDs. Participant compensation varied by recruitment source. Clinic participants received no compensation as they completed the necessary measures as part of routine care. Participants drawn from one of our team’s neurobiology of ARFID study received up to $550 (because the broader study included several multi-hour visits over a period of two years, including not just questionnaires but also fMRI scans, test meals, and blood draws). MTurk participants received $1 for completing a pre-screener and $15 for completing a battery of questionnaires.

### Measures

#### Demographics

All participants reported their age, sex, race, and ethnicity on a standard self-report form or via clinical interview.

#### Pica, ARFID and rumination disorder interview ARFID questionnaire (PARDI-AR-Q)

All participants completed the PARDI-AR-Q, which asks individuals to report on their own symptoms. (A parent version in which parents report on their child’s symptoms is also under study but was not investigated in the current study). The full 32-item questionnaire and scoring algorithm appear in Additional file 2 as well as online at mccaed.slam.nhs.uk and are freely available for use.

The PARDI-AR-Q begins with five general demographic and anthropomorphic questions. The anthropomorphic items are included so that the assessor can calculate body mass index for adults or body mass index percentile for youth (when combined with demographic data), if desired. Next, 12 dichotomous (yes/no) items assess the presence of *DSM-5* diagnostic criteria for ARFID, including the presence of an eating problem (two items), low weight or faltering growth (five items), nutrition deficiency (one item), supplement dependence (three items), and psychosocial impairment (one item). Given that several diagnostic criteria for ARFID are polythetic, the PARDI-AR-Q includes multiple items per criterion for comprehensiveness. For example, criterion A1 can be met by endorsing items 8, 9, 11, 12, and/or 13. Of note, four of the dichotomous diagnostic items include free-text follow-up questions (e.g., amount of weight lost, type of supplements used) to provide additional context for the assessor to confirm why and how specific diagnostic criteria are being fulfilled. Based on the pattern of response on these yes/no items, respondents can score positive or negative for possible ARFID based on the PARDI-AR-Q diagnostic algorithm (see Additional file 3 for scoring). “Yes” ratings should be followed up with a clinical interview to confirm diagnosis. Finally, the PARDI-AR-Q presents 11 Likert-scale items (scored 0 to 6, with higher scores indicating greater severity) designed to assess the presence and severity of each ARFID profile (i.e., avoidance based on sensory characteristics of food, lack of interest, concern about aversive consequences) as well as severity of impact.

#### Pica, ARFID and rumination disorder interview (PARDI)

A subset of participants with ARFID only (*n* = 27, 93%) also completed the PARDI [8], a structured interview designed to confirm the presence of an ARFID diagnosis as well as to provide a detailed assessment of the severity of each ARFID profile (i.e., avoidance based on sensory characteristics of food, lack of interest, or concern about aversive consequences) and the severity of overall impact. Like the PARDI-AR-Q, some questions on the PARDI were yes/no questions with follow-up questions asking the interviewee to elaborate on their response, while others were rated on a Likert scale of 0 to 6, where 6 represented the highest level of symptom severity for that question.

We scored the PARDI according to updated guidelines from a recent psychometric paper [21]. In the current study, we used the PARDI to (1) confer ARFID diagnoses among participants from the neurobiology study; and (2) evaluate concurrent validity of the three PARDI-AR-Q subscales and the severity score by assessing their degree of correlation with comparable subscales on the PARDI interview. Inter-rater reliability of the ARFID diagnosis in randomly selected subsets of the neurobiology sample have been reported previously (100% agreement; kappa = 1.0 [22, 23]). Cronbach alphas for each of the three subscales in the current sample were 0.81 (avoidance based on the sensory characteristics of food), 0.88 (lack of interest in food or eating), 0.88 (concern about aversive consequences).

#### Nine-item ARFID screen (NIAS)

A subset of participants (15 [36% of] ARFID and 26 [90% of] HC) completed the NIAS [10], a 9-item self-report questionnaire designed to measure each of the three ARFID phenotypes described in *DSM-5* including “picky eating” (3 items), “low appetite” (3 items), and "fear" (3 items). All items in the NIAS are rated on a 5-point Likert scale from 0 (“Strongly disagree”) to 5 (“Strongly agree”). The NIAS was originally validated for use in adults ages 18 and up but has also been used in adolescents [11]. In the current study we used the NIAS to evaluate the convergent validity of PARDI-AR-Q subscales. Cronbach alphas in our sample were: for the whole sample: 0.89 for picky eating, 0.91 for low appetite, and 0.92 for fear; for the ARFID sample: 0.31 for picky eating, 0.83 for low appetite, and.88 for fear; and for HC: 0.65 for picky eating, 0.71 for low appetite, and 0.82 for fear.

#### Food neophobia scale (FNS)

A subset of participants (28 [66% of] ARFID and 26 [90% of] HC) completed the FNS [24], a self-report measure of reluctance to try novel foods. All items in the FNS are rated on a 7-point Likert scale from “Disagree extremely” to “Agree extremely”, where “Agree extremely” represented the greatest severity in some items and the least severity in other, reverse-scored items. The FNS was originally validated for use in adults ages 18 and up but has also been used in adolescents [22]. In the current study, we used the FNS to evaluate convergent validity with the PARDI-AR-Q avoidance based on sensory characteristics subscale. Cronbach alphas in our sample were: 0.97 for the whole sample, 0.84 for the ARFID sample, and 0.94 for HC.

#### Clinical impairment assessment (CIA)

All participants with ARFID and 26 (90% of) HC completed the CIA [25], a self-report measure of the functional impairment associated with eating disorders. All CIA items are rated on a 4-point Likert scale from “Not at all” to “A lot”, where “A lot” represented the greatest severity. Although the CIA has not yet been validated for use with ARFID specifically, we evaluated the degree to which it was correlated with the PARDI-AR-Q severity of impact score, as a proxy measure of convergent validity. Cronbach alphas in our sample were: 0.96 for the whole sample, 0.95 for the ARFID sample, and 0.84 for HC.

#### Eating disorder examination-questionnaire (EDE-Q)

All participants in both the ARFID and HC groups completed the EDE-Q [2], which measures the specific psychopathology of eating disorders—particularly anorexia nervosa, bulimia nervosa, and binge-eating disorder—including frequency of key symptoms as well as dietary restraint, eating concern, weight concern, and shape concerns. All items in the EDE-Q are rated on a 7-point Likert scale from 0 (“No days” or “Not at all”) to 6 (“Every day” or “Markedly”), where 6 represented the greatest severity. In the current study, we used a cut-off score of 2.3 on the EDE-Q [26] to rule out other eating disorders (apart from ARFID) in both the ARFID and HC groups. Cronbach alphas in our sample were: 0.85 for the whole sample, 0.93 for the ARFID sample, and 0.71 for HC.

### Data analysis

We conducted all analyses in R [27]. First, to test our hypothesis that we would identify three PARDI-AR-Q factors reflecting avoidance based on sensory characteristics of food, lack of interest, and concern about aversive consequences, we conducted an exploratory factor analysis (EFA) of the continuously scored items that were intended to be comparable to the three subscales in the PARDI (i.e., items 24–32). Our rationale for subjecting just the nine ARFID presentation items to the primary EFA was two-fold. First, severity of impact varies across ARFID presentations. Thus, we expected the severity of impact items to correlate with all other items on the PARDI-AR-Q. Second, our team’s recently published factor analysis of the PARDI interview (Cooper-Vince et al., 2022) also analyzed only ARFID presentation items together in a single EFA; thus we took a similar analytic approach here to enhance the comparability of psychometric properties for the interview and self-report measures. (However, Additional file 1: Table S1 provides the results of an EFA that includes the severity of impact items as well.) Given that ARFID presentations often overlap clinically [28, 29], we used a promax rotation that allowed factors to inter-correlate. Given our expectation that the HC would show very low variance on these items, we included only the ARFID group in this analysis. We had no missing PARDI-AR-Q data at the participant or item level. Although the sample size for EFA was modest, the Kaiser–Meyer–Olkin measure of sampling adequacy was > 0.80 (i.e., 0.82 in our sample), and the Bartlett’s test of sphericity was significant (*X*^2^ [36] = 250.33, *p* < 0.001), suggesting that EFA was appropriate. To evaluate the internal consistency of each subscale, we calculated Cronbach alphas for each of the ARFID-only group, the HC-only group, and the full sample.

Second, to evaluate convergent validity, we evaluated the strength of correlations between PARDI-AR-Q subscales and related constructs, including the corresponding NIAS subscales, the FNS, and the CIA. To evaluate divergent validity, we evaluated the strength of correlation with EDE-Q, which should not be conceptually related to PARDI-AR-Q scores as weight and shape concerns are not central to the ARFID diagnosis. To evaluate concurrent validity, in the subset of individuals with ARFID for whom PARDI interview data were also available, we evaluated the strength of intraclass correlation coefficients between subscale scores on the PARDI-AR-Q and their analogous scores on the PARDI interview.

Third, to test our hypothesis that individuals with ARFID would score significantly higher than HC on all PARDI-AR-Q subscales, we conducted a Mann–Whitney-Wilcoxon test (given the non-normal distribution of subscale scores). We also calculated Wilcoxon *r* values to determine effect size [30]. Wilcoxon *r* effect sizes range from 0 to 1, with *r* < 0.3 indicating a small effect, 0.3–0.5 indicating a moderate effect, and > 0.5 indicating a large effect.

Lastly, we evaluated the percentage of participants with ARFID as well as HC who scored positive versus negative on the PARDI-AR-Q diagnostic algorithm. We then qualitatively reviewed responses of ARFID participants who scored negatively as well as HC who scored positively to evaluate the reasons for any discrepancies.

## Results

### Exploratory factor analysis (EFA)

The EFA identified either a three-factor solution (explaining 75% of the variance) or four-factor solution (explaining 76% of the variance) as optimal. Additional file 1: Fig. S1 depicts the scree plot. Because the three-factor solution was more interpretable and the difference in variance explained between the three- and four-factor solution was minimal, we moved forward with interpretation of the three-factor solution. Additionally, Horn’s parallel analysis provided empirical support for the retention of three factors. Table 2 displays loadings for each item on each of the three factors. Factor 1 explained 38% of the variance (eigenvalue = 22.02) and included all items assessing concern about aversive consequences (items 30–32). Factor 2 explained 34% of the variance (eigenvalue = 7.93) and included all items assessing sensory-based avoidance (items 24–26). Factor 3 explained 28% of the variance (eigenvalue = 4.64) and included all items assessing lack of interest in eating or food (items 27–29). (Of note, as depicted in Additional file 1: Table S1, adding the PARDI-AR-Q severity of impact items resulted in a 4-factor solution including concern about aversive consequences; sensory-based avoidance; lack of interest in eating or food; and severity of impact).

**Table 2:** Loadings of individual items in an exploratory analysis of the Pica, ARFID, and Rumination Disorder ARFID Questionnaire (PARDI-AR-Q) among individuals with ARFID

	Factor 1	Factor 2	Factor 3
Eigenvalue (variance explained)	22.02 (38%)	7.93 (34%)	4.64 (28%)
Item 24—Sensitivity to taste	**0.07**	**0.93**	− 0.12
Item 25—Sensitivity to texture or consistency	**– 0.14 **	**0.87**	0.05
Item 26—Sensitivity to the appearance of food	**0.09**	**0.71**	0.14
Item 27—Forgotten to eat or difficult to make time	0.06	– 0.14	**0.88**
Item 28—Lacked enjoyment in food or eating	0.10	0.18	**0.60**
Item 29—Felt full or stopped eating early	– 0.13	0.08	**0.81**
Item 30—Afraid something bad might happen	**0.93**	**– 0.20**	0.12
Item 31—Avoided eating situations due to worry	**0.90**	**0.09**	− 0.03
Item 32—Physical feelings of panic or anxiety	**0.91**	**0.13**	− 0.11

Factors on which items loaded most highly are bolded

Cronbach alphas for all subscales were good to excellent for the ARFID group (but lower for the HC group due to low variance in HC responses) for sensory-based avoidance (0.94 whole sample; 0.89 ARFID only; 0.40 HC only), concern about aversive consequences (0.92 whole sample; 0.93 ARFID only; 0.40 HC only), and lack of interest in eating or food (0.84 whole sample; 0.83 ARFID only; 0.30 HC only).

### Convergent, divergent, and concurrent validity

Table 3 depicts a Spearman correlation matrix with each of the three PARDI-AR-Q subscales and measures of convergent (i.e., NIAS subscales, FNS) and divergent (EDE-Q) validity in the whole sample. As hypothesized, supporting convergent validity, PARDI-AR-Q sensory-based avoidance was highly correlated with NIAS picky eating and with the FNS; PARDI-AR-Q concern about aversive consequences was highly correlated with NIAS fear; and PARDI-AR-Q lack of interest in food or eating was highly correlated with NIAS low appetite. Also as anticipated, all PARDI-AR-Q subscales were correlated with one another, and with all NIAS subscales. Lastly, PARDI-AR-Q severity of impact was highly correlated with CIA scores.

**Table 3:** Spearman correlation matrix of scores on the Pica, ARFID, and rumination disorder interview ARFID questionnaire (PARDI-AR-Q) and related measures for the whole sample

	SBA	LOI	FAC	SOI	FNS	PE	LA	F	EDE-Q	CIA
SBA	1.0	0.68**	0.43**	0.79**	0.75**	0.7**	0.69**	0.48**	− 0.15	0.62**
LOI		1.0	0.54**	0.70**	0.63**	0.49**	0.76**	0.53**	0.20	0.72**
FAC			1.0	0.42**	0.41**	0.48**	0.42**	0.65**	0.18	0.50**
SOI				1.0	0.69**	0.48**	0.61**	0.39*	0.09	0.75**
FNS					1.0	0.89**	0.53**	0.50**	− 0.04	0.61**
PE						1.0	0.49**	0.44**	− 0.04	0.47**
LA							1.0	0.67**	0.02	0.58**
F								1.0	− 0.18	0.43**
EDE-Q									1.0	0.27*
CIA										1.0

All participants completed the PARDI-AR-Q and EDE-Q, so correlations among SBA, LOI, FAC, and EDE-Q are based on the full data set. Because only a subsample completed FNS (n = 54) and NIAS (n = 41), FNS, PE, LA, and F correlations including those variables reflect the subset only

*SBA* PARDI-AR-Q sensory-based avoidance; *LOI* PARDI-AR-Q lack of interest in eating or food; *FAC* PARDI-AR-Q concern about aversive consequences; *SOI* PARDI-AR-Q severity of impact; *FNS* food neophobia scale; *PE* nine-item ARFID screen (NIAS) picky eating; *LA* NIAS low appetite; *F* NIAS fear; *EDE-Q* eating disorder examination-questionnaire global score

*p < 0.05

**p < 0.01

Furthermore, as hypothesized, none of the PARDI-AR-Q subscales were significantly correlated with EDE-Q global score (which is intended to measure a different construct, i.e., purposeful dietary restraint and shape/weight concerns). In the subset of ARFID participants who also completed the PARDI interview (*n* = 27), intraclass correlations were large and significant across subscales, supporting concurrent validity (avoidance based on sensory characteristics of food: *r* = 0.73, *p* < 0.001; concern about aversive consequences: *r* = 0.54, *p* < 0.001; lack of interest: *r* = 0.73, *p* < 0.001; and severity of impact: *r* = 0.48, *p* < 0.001).

### Comparisons between ARFID and HC

Table 4 displays PARDI-AR-Q subscale scores in the ARFID group versus HC. As hypothesized, the ARFID group scored significantly higher than HC on all subscales. All but one effect size was large. Concern about aversive consequences had a medium effect size. Given that the two groups differed significantly by sex and age (Table 1), we followed this up with an ANCOVA controlling for sex and age (Additional file 1: Table S2), and results remained unchanged.

**Table 4:** Scores (*M*, *SD*) on the Pica, ARFID, and Rumination Disorder Interview Questionnaire (PARDI-AR-Q) and relevant measures for individuals with avoidant/restrictive food intake disorder (ARFID) versus the healthy control participants (HC)

	ARFID *n = 42	HC *n = 29	Test statistic (*W* or *X*^2^)	Effect size (Wilcoxon *r*)	*p*
PARDI-AR-Q Sensory-based avoidance	3.11 (2.07)	0.05 (0.11)	1119	0.72	< 0.001
PARDI-AR-Q Lack of interest in eating or food	2.65 (1.70)	0.62 (0.78)	1017	0.55	< 0.001
PARDI-AR-Q Concern about aversive consequences	1.48 (2.04)	0.24 (0.58)	819.5	0.33	< 0.01
PARDI-AR-Q Severity of impact	2.35 (1.92)	0.07 (0.18)	1051	0.63	< 0.001
Food neophobia scale	60.25 (9.15)	27.85 (13.92)	697.5	0.66	< 0.001
NIAS picky eating	13.07 (2.12)	3.69 (2.74)	388	0.60	< 0.001
NIAS low appetite	8.8 (4.30)	1.46 (2.04)	356.5	0.51	< 0.001
NIAS fear	6.13 (4.97)	0.62 (1.13)	322	0.44	< 0.001
EDE-Q global	0.60 (0.77)	0.79 (0.67)	492	− 0.16	0.17
CIA	13.52 (11.99)	2.42 (3.05)	885.5	0.49	< 0.001

*M* mean; *SD* standard deviation; *NIAS* Nine-Item ARFID Scale; *EDE-Q* Eating disorder Examination-Questionnaire; *CIA* Clinical Impairment Assessment

*In the ARFID sample, *n* = 28 participants completed the Food Neophobia Scale, and *n* = 15 participants completed the Nine-Item ARFID Screen; in the HC group, *n* = 26 participants completed the Food Neophobia Scale data, and *n* = 26 completed the Nine-Item ARFID Screen

### Positive and negative scores on the PARDI-AR-Q diagnostic algorithm in ARFID vs. HC

Of the 42 participants in the ARFID group, 90% (n = 38) scored positive on the PARDI-AR-Q diagnostic algorithm. However, the remaining 10% (n = 4) scored negative on the diagnostic algorithm, despite receiving an ARFID diagnosis via PARDI or clinical interview. Of the four who scored negative, one was an adolescent male who met criteria via interview based on having an underweight BMI percentile and psychosocial impairment (diagnostic criteria A1 and A4, respectively). This participant was also undergoing psychological treatment for ARFID at the time. However, this participant did not endorse any items on the PARDI-AR-Q. The other three false negative cases met criteria for ARFID during the interview based on psychosocial impairment only (diagnostic criterion A4). However, all three scored negative on the PARDI-AR-Q because they did not endorse item 21 (psychosocial impairment), though two of the three did endorse items 6 (thinking they had a problem with eating) and 7 (other people saying they had a problem with eating).

In contrast, of the 29 of participants in the HC group, only 7% (n = 2) scored positive on the PARDI-AR-Q diagnostic algorithm, while 93% (n = 27) scored negative. Both HC participants who scored positive on the diagnostic algorithm endorsed item 6 (thinking they had a problem with eating) and 9 (weight loss due to avoidance or restriction). Both participants were adults with BMIs in the overweight range, based on the heights and weights they provided on the PARDI-AR-Q.

## Discussion

Though replication in larger and more diverse samples is needed, findings provide early support for the validity and reliability of the PARDI-AR-Q as a self-report measure of possible ARFID in clinical or research settings. Specifically, our exploratory factor analysis provided preliminary support for a three-factor structure consistent with *DSM-5* ARFID phenotypes. Furthermore, medium to large correlations between PARDI-AR-Q subscales and related constructs (e.g., NIAS, FNS, CIA) provided preliminary support for convergent validity, and high intraclass correlations between PARDI-AR-Q subscales and their corresponding subscales on the PARDI-AR-Q provided preliminary evidence of concurrent validity. In contrast, low and non-significant correlations between PARDI-AR-Q subscales and EDE-Q global scores provided preliminary support for divergent validity. Finally, as expected, the majority of participants with ARFID scored positive on the PARDI-AR-Q whereas the majority of the HC scored negative.

PARDI-AR-Q subscale scores correlated highly with one another, but still loaded onto distinct factors. This was also true of NIAS subscale scores in the current study, as well as in prior studies [10]. Taken together, these findings are consistent with existing reports of overlap among ARFID phenotypes [28] and lend support to a three-dimensional model of ARFID in which there are three distinct phenotypes and individuals may present with one, two, or all three [29].

Our qualitative analysis of discordant cases highlights the challenges inherent to the self-report assessment of ARFID diagnostic criteria. In the case of the four participants diagnosed with ARFID who scored negative on the PARDI-AR-Q diagnostic algorithm, one participant was underweight and concurrently undergoing ARFID treatment. This patient may have exhibited a minimizing response style on the questionnaire, which is also common in other eating disorders, such as anorexia nervosa [31]. In the other three cases, individuals denied psychosocial impairment as specifically queried on the PARDI but were ascertained by their PARDI interviewer or treating clinician to have eating-related impairment present. Given that currently available measures of functional impairment due to disordered eating have not been validated for ARFID specifically, a better understanding of the impairing features unique to ARFID (versus other eating disorders) is urgently needed, to ensure that questionnaires such as the PARDI-AR-Q can fully capture this impairment. In the case of the two HC participants with positive PARDI-AR-Q scores, both endorsed restrictive eating in the context of overweight. It is unclear whether they would have met criterion A1 upon clinical interview.

Our findings should be interpreted in light of limitations. Our sample size was modest, and our sample was from a single country (United States) and identified primarily as non-Hispanic white, thus limiting generalizability. This limitation is particularly important in the context of assessment, which may play a gate-keeping function for treatment access. Future research on the PARDI-AR-Q and other eating-disorder assessments should recruit more racially, ethnically, and geographically diverse samples [32]. Similarly, we did not include participants under the age of 14 in the current study, so our findings cannot be generalized to children. (Our team is currently working on a parent-report version of the PARDI-AR-Q for this younger age range.) In addition, the HC group did not complete the PARDI or routine clinical interview, but rather were characterized as HC by their non-clinical scores on a battery of self-report questionnaires; and not all ARFID participants completed the PARDI interview as some were evaluated in a routine clinical setting. To that end, we could not calculate sensitivity and specificity in the current study. Moreover, there are challenges to collecting psychological data through MTurk (e.g., difficulty confirming that participants have represented demographic characteristics truthfully and were paying attention to all survey questions), which may have impacted the HC group for this study. Lastly, because the Cronbach alpha value for HC on all PARDI-AR-Q subscales and the Cronbach alpha value for ARFID on the NIAS picky eating scale were fairly low, more research is needed on how measures of ARFID psychopathology may differentially perform in ARFID vs. HC populations, and how that may impact comparisons between these groups. Of note, our HC group included only those individuals who scored below established clinical cut-offs on measures of depression, anxiety, and disordered eating; or showed no evidence of psychiatric disorder on the KSADS. Indeed, as shown in Table 4, our HC group scored near the minimum on nearly every measure of psychopathology, including the NIAS, EDE-Q, and CIA. These findings suggest that that the low PARDI-AR-Q scores in our HC group may be reflective of their especially their low levels of psychopathology, rather than the PARDI-AR-Q’s inability to adequately capture ARFID symptoms in a non-clinical group. However, future research is needed to clarify normative PARDI-AR-Q scores in community samples that include the full range of psychopathology.

The study also had strengths. Specifically, our entire ARFID sample included participants whose diagnosis had been confirmed via PARDI or structured clinical interview. This is particularly important as past studies of self-report measures of ARFID have largely relied on analogue or college samples with picky eating [10]. Further efforts to validate and improve upon the PARDI-AR-Q should include larger and more diverse samples, all of whom are followed up by clinical interview to confirm diagnosis.

## Conclusion

In summary, the PARDI-AR-Q showed preliminary evidence of reliability and validity in the current study. The PARDI-AR-Q has advantages over existing self-report and interview measures of ARFID symptoms. For example, while the PARDI-AR-Q and NIAS both measure ARFID psychopathology via self-report, only the PARDI-AR-Q has a severity of impact scale and diagnostic algorithm for screening purposes. Indeed, in the current study, the PARDI-AR-Q correctly identified 90% of ARFID cases, regardless of specific ARFID profile, based on *DSM-5* criteria. Compared to clinical interviews (e.g., PARDI, EDA-5, SCID-5), the PARDI-AR-Q is quick to administer and does not require a trained interviewer or clinician for scoring. Our findings suggest that the PARDI-AR-Q can be used to elucidate clinical characteristics (e.g., profiles) when a clinical interview is not practical or desirable. Future research should use the PARDI as part of a two-stage screening process with which to evaluate the diagnostic properties of the PARDI-AR-Q against a gold standard interview.

## Prior presentation

Portions of these findings were presented at the 2020 Eating Disorders Research Society meeting and 2022 International Conference on Eating Disorders (both held virtually due to COVID-19).

## Supplementary Information

**Additional file 1.** Supplemental tables and supplemental figure.

**Additional file 2**. PARDI-AR-Q Self 14+.

**Additional file 3.** PARDI-AR-Q instructions for administrators.

## Data Availability

The data that support the findings of this study are available from the corresponding author upon reasonable request.
