# Supplementary material for: Preliminary validation of the pica, ARFID and rumination disorder interview ARFID questionnaire (PARDI-AR-Q)
Source: J Eat Disord. 2022 Nov 22;10:179. doi: 10.1186/s40337-022-00706-7 (PMC9682666; doi:10.1186/s40337-022-00706-7)

Supplemental Table 1: Loadings of individual items in an exploratory factor analysis of the Pica, ARFID, and Rumination Disorder ARFID Questionnaire (PARDI-AR-Q) among individuals with ARFID, including the two severity items in the PARDI-AR-Q and identifying an alternative four-factor solution with severity of impact being its own factor.

|  | Factor 1 | Factor 2 | Factor 3 | Factor 4 |
| --- | --- | --- | --- | --- |
| Eigenvalue (variance explained) | 2.78 (18%) | 26.37 (31%) | 8.36 (28%) | 4.89 (23%) |
| Item 22—Severity item: relational difficulties | **1.0** | -.07 | -.12 | .09 |
| Item 23 — Severity item: social situations | **.55** | .01 | .14 | .19 |
| Item 24—Sensitivity to taste | .03 | .08 | **.86** | -.12 |
| Item 25—Sensitivity to texture or consistency | -.23 | -.12 | **1.0** | .12 |
| Item 26—Sensitivity to the appearance of food | .22 | .08 | **.66** | -.01 |
| Item 27—Forgotten to eat or difficult to make time | -.01 | .06 | -.15 | **.96** |
| Item 28—Lacked enjoyment in food or eating | .11 | .11 | .14 | **.54** |
| Item 29—Felt full or stopped eating early | .04 | -.09 | .13 | **.68** |
| Item 30—Afraid something bad might happen | -.19 | **.96** | -.12 | .19 |
| Item 31—Avoided eating situations due to worry | -.01 | **.90** | .09 | -.02 |
| Item 32—Physical feelings of panic or anxiety | -.15 | **.91** | .04 | -.16 |

*Note*. Factors on which items loaded most highly are **bolded.**

Supplemental Table 2. Scores (*M*, *SD*) on the Pica, ARFID, and Rumination Disorder Interview Questionnaire (PARDI-AR-Q) for individuals with avoidant/restrictive food intake disorder (ARFID) versus the healthy control participants (HC) in Analyses of Covariance (ANCOVA) controlling for sex and age.

|  | ARFID  **n* = 42 | HC  **n* = 29 | Test statistic  (*F*) | Effect size  (*d*) | *p* |
| --- | --- | --- | --- | --- | --- |
| **Sensory-based avoidance** | **3.11 (2.07)** | **0.05 (0.11)** | **31.51** | **1.97** | **< .001** |
| **Lack of interest in eating or food** | **2.65 (1.70)** | **0.62 (0.78)** | **25.84** | **1.43** | **< .001** |
| **Concern about aversive consequences** | **1.48 (2.04)** | **0.24 (0.58)** | **6.08** | **0.72** | **< .05** |
| **Severity of impact** | **2.35 (1.92)** | **0.07 (0.18)** | **24.67** | **1.53** | **< .001** |
| Food Neophobia Scale | 60.25 (9.25) | 27.85 (13.92) | 64.80 | 2.77 | <.001 |
| NIAS Picky Eating | 13.07 (2.12) | 3.69 (2.74) | 68.04 | 3.70 | <.001 |
| NIAS Low Appetite | 8.8 (4.3) | 1.46 (2.04) | 33.29 | 2.41 | <.001 |
| NIAS Fear | 6.13 (4.97) | 0.62 (1.13) | 17.51 | 1.77 | <.001 |
| EDE-Q Global | 0.60 (0.77) | 0.79 (0.67) | 0.57 | -0.26 | 0.45 |
| CIA | 13.52 (11.99) | 2.42 (3.05) | 14.23 | 1.15 | <.001 |

Note. *M* = mean; *SD* = standard deviation; NIAS = Nine-Item ARFID Scale; EDE-Q = Eating Disorder Examination-Questionnaire; CIA = Clinical Impairment Assessment.

*In the ARFID sample, *n* = 28 participants completed the Food Neophobia Scale, and *n* = 15 participants completed the Nine-Item ARFID Screen; in the HC group, *n* = 26 participants completed the Food Neophobia Scale data, and *n* = 26 completed the Nine-Item ARFID Screen.

Supplemental Figure 1.

Scree plot visualizing exploratory factors and eigenvalues for 9 continuously-scored items in the Pica, ARFID, and Rumination Disorder ARFID Questionnaire (PARDI-AR-Q) among individuals with ARFID.
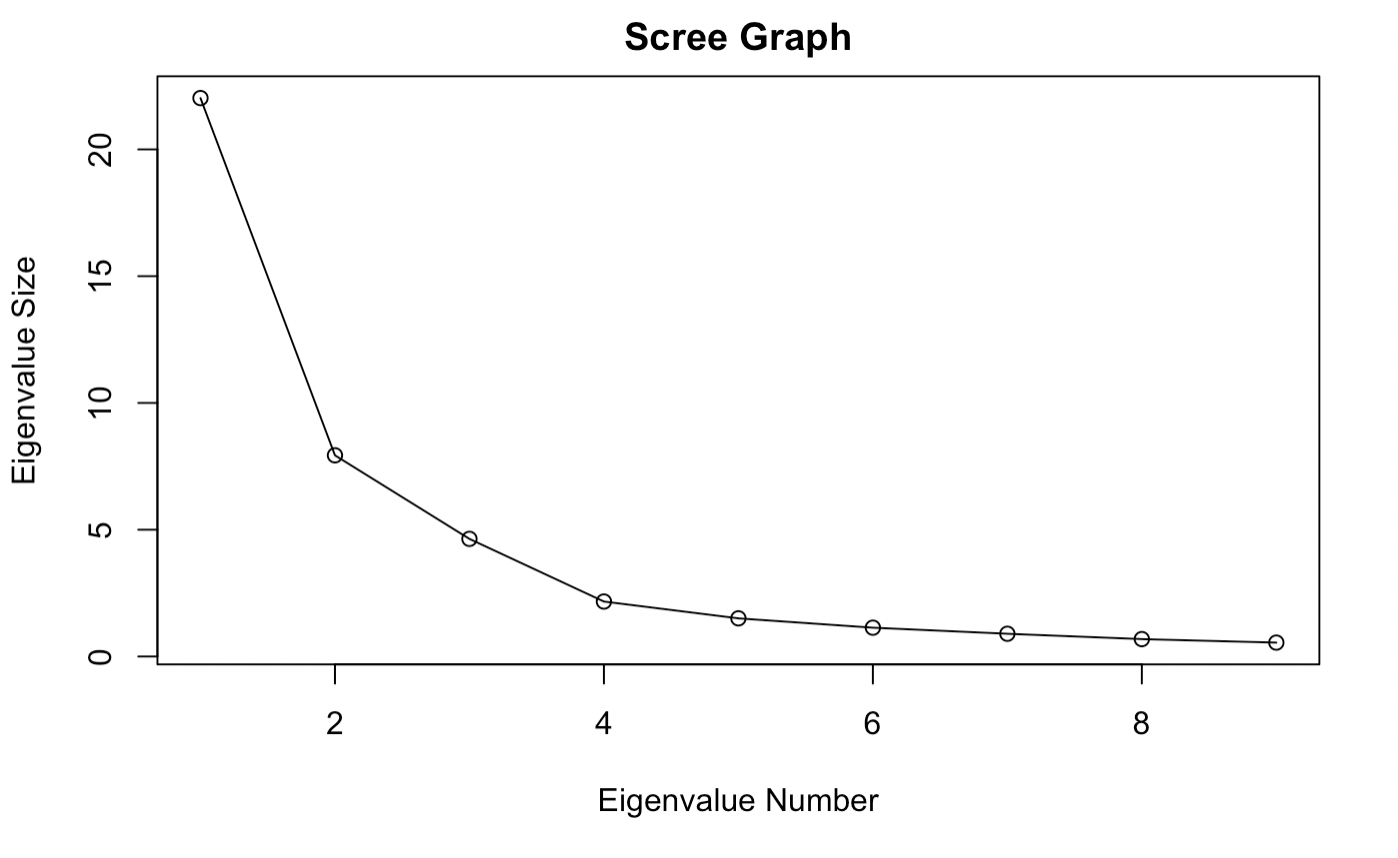

Supplement: Supplementary file 1 — Additional file 1. Supplemental tables and supplemental figure. [file 40337_2022_706_MOESM1_ESM.docx]
