# Supplementary material for: Preliminary validation of the pica, ARFID and rumination disorder interview ARFID questionnaire (PARDI-AR-Q)
Source: J Eat Disord. 2022 Nov 22;10:179. doi: 10.1186/s40337-022-00706-7 (PMC9682666; doi:10.1186/s40337-022-00706-7)
Supplement: Supplementary file 2 — Additional file 2. PARDI-AR-Q Self 14+. [file 40337_2022_706_MOESM2_ESM.pdf]

For office use - ID:

PARDI-AR-Q: Self 14+

The following questions are about your eating – some ask about how things currently are, others ask about things over the past month or the past 3 months. Please tick the boxes that apply to you, or enter the information requested. **Please read each question carefully. Please answer all the questions. Thank you.**

1. Please fill in today's date: ____/______/_______ (day/month/year)

2. Please fill in your date of birth: ____/______/_______ (day/month/year)

3. Are you? Male ☐ Female ☐ Other ☐ _______________

4. What is your current height? (please enter numbers): feet ☐ in ☐ **/OR** metres ☐ cm ☐

5. What is your current weight? (please enter numbers): lbs ☐ **/OR** stones ☐ lbs ☐ **/OR** kg ☐

6. Do you think you have a problem with eating, involving avoidance or restriction of foods or your eating overall? Yes ☐ No ☐

7. Have other people (for example, doctors, family members, significant others) said that you have a problem with eating, involving avoidance or restriction of foods or your eating overall? Yes ☐ No ☐

8. Have your eating habits led to difficulty maintaining a sufficient weight or, if you are still growing, difficulty gaining enough weight to keep pace with your growth? Yes ☐ No ☐

9. Have your eating habits led to you losing weight (in other words, if you have lost weight, this is because of avoidance or restriction and not because of a medical illness, or other reason)? Yes ☐ No ☐

10. If yes to #9 above, how much weight have you lost in the past 3 months? (please enter numbers): lbs ☐ **/OR** stones ☐ lbs ☐ **/OR** kg ☐ **OR** No weight loss over past 3 months ☐

11. Have others (for example, doctors, family members) been concerned about your weight loss, or been concerned that you are having difficulty gaining enough weight to grow, or having difficulty maintaining your weight due to your eating habits? Yes ☐ No ☐

12. Have others (for example, doctors, family members) been concerned that you are not growing taller as you should due to your eating habits? Yes ☐ No ☐ I have finished growing ☐

13. Have you <u>ever</u> been told by **any health professional** that due to your eating habits you were not growing as expected, or that your height was less than it should be? Yes ☐ No ☐

14. Over the past month, has **any health professional** said that you have a nutritional deficiency due to your eating habits (for example, low iron, low vitamin B12, low vitamin C)? Yes ☐ No ☐

©Rachel Bryant-Waugh, Kamryn Eddy, Nadia Micali, Lucy Cooke, Jennifer J. Thomas

For office use - ID:

15. Over the past month, has **a healthcare professional prescribed** special supplements (for example, pills, capsules, powders, or drinks containing vitamins and/or minerals and other micronutrients) **specifically to help with your nutrition?** Yes ☐ No ☐

16. If yes to #15 above, what have you been prescribed and how much do you take each day?

____

17. Over the past month, has **a healthcare professional prescribed** special supplements (for example, high-calorie drinks or 'shots', or dessert-style high-calorie supplements) **specifically to help you maintain or gain weight?** Yes ☐ No ☐

18. If yes to #17 above, what have you been prescribed and how much do you take each day?

____

19. Are you currently receiving any tube feeding (receiving food or fluid via a tube in your nose or into your stomach)? Yes ☐ No ☐

20. If yes to #19 above, what is the name of the **food or fluid product you take via the tube** and how much do you take each day?

____

21. Does your eating cause you difficulties in daily functioning - that is, in how you are able to go about things each day? This might be at school/college/work or when you are at home. Yes ☐ No ☐

22. Does your eating cause you difficulties in interactions with other people (for example, disagreements or arguments with parents, siblings, significant others, co-workers), or difficulty making or sustaining friendships or other close relationships?
Please circle a number on the line below how difficult **interactions with other people** are for you because of your eating, ranging from 0 (= no difficulty) to 6 (= extremely difficult)

0 1 2 3 4 5 6 →

23. Does your eating cause you difficulties in social situations, for example does it make it difficult for you to go out with friends, eat at school/college/work, or stay away from home?

Please circle a number on the line below how difficult **social situations** are for you because of your eating, ranging from 0 (= no difficulty) to 6 (= extreme difficulties/try to avoid all social situations)

0 1 2 3 4 5 6 →

24. Over the past month, have you been particularly sensitive to variation in taste (for example, noticing slight differences in the taste of foods), which has put you off eating any foods or trying any new foods?

Please circle a number on the line below how much **sensitivity to taste** has affected your eating, ranging from 0 (= no negative effect/no particular sensitivity to taste) to 6 (= extremely negative effect, for example, leading to refusal to eat many foods, sticking only to a limited number of preferred foods, or extreme caution when eating)

0 1 2 3 4 5 6 →

©Rachel Bryant-Waugh, Kamryn Eddy, Nadia Micali, Lucy Cooke, Jennifer J. Thomas

25. Over the past month have you been particularly sensitive to the texture or consistency of food, which has put you off eating any foods or trying any new foods (for example, do you stick to foods of a certain texture only or have you had difficulty eating foods that have different textures mixed together such as pasta with sauce or sandwiches)?

Please circle a number on the line below how much **sensitivity to texture or consistency** has affected your eating, ranging from 0 (= no negative effect/no particular sensitivity) to 6 (=extremely negative effect, for example, leading to refusal to eat many foods, sticking only to a limited number of preferred foods, or extreme caution when eating)

0 1 2 3 4 5 6

26. Over the past month, have you been particularly sensitive to the appearance of food, which has put you off eating any foods or trying any new foods (for example, if food does not look "right", such as burnt ends of chips/fries, broken biscuits/cookies, or being the "wrong" colour)?

Please circle a number on the line below how much **sensitivity to the appearance of food** has affected your eating, ranging from 0 (= no negative effect/no particular sensitivity) to 6 (=extremely negative effect, for example, leading to refusal to eat many foods, sticking only to a limited number of preferred foods, or extreme caution when eating)

0 1 2 3 4 5 6

27. Over the past month, how often have you forgotten to eat or found it difficult to make time to eat? Please circle a number of the line below how often you have **forgotten to eat or found it difficult to make time to eat**, ranging from 0 (= never) to 6 (=always)

0 1 2 3 4 5 6

28. Over the past month, how often have you lacked enjoyment in food or eating (even if only certain foods)?
Please circle a number on the line below how often you **have lacked enjoyment in food or eating**, ranging from 0 (= never) to 6 (=always)

0 1 2 3 4 5 6

29. Over the past month, how often have you felt full before your meal is finished, or stopped eating sooner than others because you had had enough?

Please circle a number on the line below how often you have **felt full or stopped eating early**, ranging from 0 (= never) to 6 (=always)

0 1 2 3 4 5 6

30. Over the past month have you been avoiding or restricting the amount or type of food you eat, because you were afraid that something bad might happen, like being sick, choking, having an allergic reaction, or being in pain?

Please circle a number on the line below how often being **afraid something bad might happen** has affected your eating, ranging from 0 (= never) to 6 (=always)

0 1 2 3 4 5 6

©Rachel Bryant-Waugh, Kamryn Eddy, Nadia Micali, Lucy Cooke, Jennifer J. Thomas

31. Over the past month have you avoided eating situations because you were worried something bad might happen, like being sick, choking, having an allergic reaction, or being in pain while eating (for example, because you might be served something you usually avoid for these reasons, or because you have had a bad experience in the past)?

Please circle a number on the line below how often you have **avoided eating situations** due to such worries, ranging from 0 (= never) to 6 (=always)

0 1 2 3 4 5 6

32. Over the past month have you had any physical feelings of panic or anxiety (examples might include a racing heart, sweaty palms, feeling sick) when you have seen something that has made you think something bad might happen, like being sick, choking, having an allergic reaction, or being in pain while eating

Please circle a number on the line below how often you have **had physical feelings of panic or anxiety** due to such thoughts, ranging from 0 (= never) to 6 (=always)

0 1 2 3 4 5 6

THANK YOU!

©Rachel Bryant-Waugh, Kamryn Eddy, Nadia Micali, Lucy Cooke, Jennifer J. Thomas
