# Supplementary material for: Preliminary validation of the pica, ARFID and rumination disorder interview ARFID questionnaire (PARDI-AR-Q)
Source: J Eat Disord. 2022 Nov 22;10:179. doi: 10.1186/s40337-022-00706-7 (PMC9682666; doi:10.1186/s40337-022-00706-7)
Supplement: Supplementary file 3 — Additional file 3. PARDI-AR-Q instructions for administrators. [file 40337_2022_706_MOESM3_ESM.pdf]

PARDI-AR-Q

Introduction for Administrators

The PARDI-AR-Q is a self-report measure of the symptoms of avoidant restrictive food intake disorder (ARFID), based on the Pica, ARFID, and Rumination Disorder Interview (PARDI; recommended citation: **Bryant-Waugh, R., Micali, N., Cooke, L., Lawson, E. A., Eddy, K. T., & Thomas, J. J. (2019).** Development of the Pica, ARFID, and Rumination Disorder Interview, a multi-informant, semi-structured interview of feeding disorders across the lifespan: A pilot study for ages 10–22. *International Journal of Eating Disorders.* 52(4):378-387)

The PARDI-AR-Q (**Bryant-Waugh, R., Eddy, K. T., Micali, N., Cooke, L., & Thomas, J. J. (2019).** The PARDI ARFID Questionnaire: a self-report multi-informant measure of the key features of avoidant restrictive food intake disorder. *Available on request from* rachel.bryant-waugh@slam.nhs.uk) can be used to predict a likely diagnosis of ARFID, which should subsequently be confirmed by clinical interview, as well as to measure the severity of impact of self-reported ARFID symptoms and ratings of common ARFID features (sensory-based avoidance; lack of interest in eating or food; and concern about aversive consequences of eating).

The PARDI-AR-Q does not include evaluation of the exclusion criteria for an ARFID diagnosis, which include the presence of other possible disorders that may account for the feeding and eating difficulty (e.g. anorexia nervosa, bulimia nervosa, or another concurrent medical condition or mental disorder that may account for the eating disturbance).

The questionnaire comes in two versions:
PARDI-AR-Q Self 14+ - suitable for young people and adults aged 14 years and over
PARDI-AR-Q Parent/carer 4+ - suitable for parents/carers of anyone over the age of 4 years

Yes responses are scored as 1 and No responses are scored as 0
Details of how to utilize responses are set out below:

Height, weight, body mass index by age and sex

ASSESSMENT AGE
Calculated by subtracting Item 2 (birth date) from Item 1 (current date)

HEIGHT CENTILE AND WEIGHT CENTILE
Calculated by using Item 3 (sex), assessment age (see above), with values for Item 4 (height) and Item 5 (weight) on standard growth charts

BODY MASS INDEX
Calculated by dividing the value in Item 5 (weight: if in lbs, or stones and lbs, needs transposing into kg), by the value in Item 4 (height: if in feet and inches needs transposing into m) squared, using the formula BMI = kg/m^2

For children and adolescents, BMI should be expressed as BMI centile, BMI Z-score, % median BMI, plotted on standard growth charts for males and females

Diagnostic predictors

Prediction of possible diagnosis of ARFID is flagged on the basis of answers in the following pattern:

Item 6 OR Item 7: Yes (1)
AND
Significant weight loss or failure to grow/ gain weight (Item 8 (1) OR Item 9 (1) OR Item 11 (1) OR Item 12 (1) OR Item 13 (1))
OR
Significant nutritional deficiency (Item 14 (1))
OR
Dependence on enteral feeding or nutritional supplements (Item 15 (1) OR Item 17 (1) OR Item 19 (1))
OR
Psychosocial impairment (Item 21 (1))

Severity of impact
Add: Item 22 and Item 23 and divide by 2

Ratings of ARFID profiles

Sensory-based avoidance – Add scores on Items 24, 25, 26 and divide by 3
Lack of interest in food and eating Add scores of items 27, 28, 29 and divide by 3
Concern about aversive consequences – Add scores on items 30, 31, 32 and divide by 3

NB: Item 10 provides contextual information to flag concerning weight loss

**Final Scores:**	
**Diagnostic prediction:**	**YES /NO**
**Severity of impact:**	**0-6**
**Sensory based avoidance:**	**0-6**
**Lack of interest:**	**0-6**
**Concern about aversive consequences:**	**0-6**
